# Don't Roll Your Manuscripts; and Other Don'ts

**Published:** 1879-10

**Authors:** 


*Don't Roll your Manuscripts; and other Don'ts.*—Some say the editors of the country will form an association pledged to read no manuscripts that have been rolled up. The mail will carry a flat package just as well as a rolled one; and the vexation of reading paper which won't come unrolled is exceeding. We have plenty of other worries without this unnecessary one.

Don't write with a pencil. It rubs out, and saves the writer no time. If you *must* write with pencil, wet the sheets in water afterwards, and carefully dry. This helps to fix the marks, and prevents erasure from slight causes. But it is better to use a pen and—*write plainly.* Illegible manuscript is one of the reserved rights of editors, which correspondents and contributors must not infringe upon. The editing is done between times, and we practice dentistry, and need our eyes.

Don't be afraid to say what you have to say in plain words. Verbosity is a useless acquirement, and is in every body's way. Short, one, two, three page articles are almost always readable. Even half a page of *original* work is worth more than some small volumes we could name.

Don't hesitate because you cannot say just in the best way your "say." If you have ideas, they are what we want; what the profession wants; what every body wants. If your way of saying it is bad, we will try and mend the way by a "revision." That's one of the offices of the editors.

Don't fail to observe closely. Dentistry must progress. But of the thousands who are at work, most are men delvers, working for money. How many of you know for yourselves that saliva is alkaline, or acid, or anything? It is a mistake to suppose that all these things are to be left to the scientist. You realize very sensibly if you reflect at all, that so far, we are practicing a very imperfectly developed science. We belong to the world's army of "healers," and suffering mankind looks to us to palliate, cure, prevent This last, how important! Most important of all! It may be a long way off, possibly even unattainable, but surely worth the striving for. A better filling material is wanted. It is surely not too much to say that chemistry can produce it, and it would be the shame of dentistry if it should come from outside the profession. There is plenty of work then. Don't rest content that you are stopping a few teeth and making artificial ones,—and money. H.

